# Transformer maintenance strategy based on improved risk assessment method considering operation and maintenance costs

**DOI:** 10.1371/journal.pone.0338610

**Published:** 2025-12-11

**Authors:** Hongbing Guo, Yan Wang, Hongbin Bo, Jianying Zhang, Yue Yang, Zhiqi Guo

**Affiliations:** 1 Inner Mongolia Power (Group) Co., Ltd., Inner Mongolia Power Research Institute Branch, Hohhot, Inner Mongolia, China; 2 School of Reliability and System Engineering, Beihang University, Beijing, China; 3 Inner Mongolia Electric Power Group Co., Ltd, Hohhot, Inner Mongolia, China; 4 Institute of Unmanned System, Beihang University, Beijing, China; Henan Polytechnic University, CHINA

## Abstract

The rapid development of modern industry cannot be separated from the support of large power equipment such as transformers. Transformers need to maintain high reliability to provide power support, and adopting a reasonable maintenance strategy is one of the important methods to maintain high reliability. At present, maintenance strategy research around transformers mainly focuses on transformer groups, single equipment or components, and lacks specialized research on specific failure modes. The overall maintenance strategy had the problem of insufficient identification and prevention of specific failure, which led to insufficient focus on resource investment. Traditional risk analysis methods insufficiently consider some risk factors and weight allocation. Therefore, this study considers specific failure modes of transformers and obtains a cost-oriented maintenance strategy for failure modes based on an improved risk analysis method. This study provides a new method for maintenance management of transformers, which helps to allocate maintenance resources reasonably and reduce maintenance costs.

## 1. Introduction

As types of large-scale power equipment, transformers need to maintain a high level of reliability [1]. Once a malfunction occurs, it can not only cause serious economic losses, but also affect the personal safety of residents. Many factors affect the reliability level of transformers [2,3], and maintenance is a crucial factor that cannot be ignored.

The complex structure of modern equipment leads to multiple failure modes [4], and current maintenance work mostly formulates maintenance strategies on the basis of the overall working status of the equipment [5–8], with transformers being no exception. These methods for determining maintenance strategies typically focus on the equipment itself and lack attention to specific failure modes. This lack may result in the inability to accurately identify and prevent critical failure modes. In this approach, safety hazards may not be identified, and the reliability and safety of the system may be negatively affected [9]. Therefore, Bayesian methods are used in this study to predict the probabilities of specific failure modes. On this basis, a risk priority method that independently considers operational and maintenance costs is used to assign weights to risk-influencing factors, set safety factors for failure modes, and determine the final recommended maintenance interval value, thus providing a reference for work by maintenance personnel.

Therefore, on the basis of the operational data, maintenance data, and expert experience with failure modes of transformers, Bayesian methods are applied to perform a reliability analysis of transformers. On the basis of the reliability analysis, operation and maintenance costs are innovatively incorporated into the risk priority calculation process, and weights are assigned to risk influencing factors through subjective-objective fusion method to improve the risk priority calculation method. Finally, on the basis of the above results, maintenance intervals for different failure modes are obtained to avoid insufficient attention and omission.

The main contributions and innovations of this research can be summarized as follows:

1) Refine the focus of maintenance research to specific failure modes, accurately identify failure, and optimize the allocation of maintenance resources;
2) Independently consider operational and maintenance costs in risk analysis to increase attention to costs;
3) Consider safety factors when calculating maintenance intervals to reduce risks.

In the second part, the current research status related to the content of this research is introduced, and the shortcomings of existing research are summarized. In the third section, the calculation methods and principles of this research, such as the Bayesian method, Markov chain Monte Carlo method, and improved risk priority number method, are discussed. In the fourth part, actual cases are explored, and the typical failure modes of 220 kV oil-immersed transformers are selected for analysis to calculate recommended maintenance strategy. Finally, the fifth section provides a summary of this research.

## 2. Related work

### 2.1. Transformer maintenance strategy

Maintenance strategies affect the reliability level of equipment. Different maintenance strategies have different impacts on the working status of equipment [10]. The calculation and optimization of maintenance intervals are important components of maintenance strategies. Conducting reliability analyses of transformers and developing maintenance strategies on the basis of the analysis results is an effective method. This method can transform the reliability analysis results from theory to practice to maintain the performance and working conditions of transformers.

At present, most research on maintenance strategies is based on historical data to analyze and calculate reliability indicators, ultimately yielding certain results. Petar Sarajcev et al. used Bayesian statistical learning methods to optimize the scheduling of transformer maintenance, in order to improve overall system reliability and reduce unplanned power outages. This article focused on transformer groups and plans the maintenance sequence of transformers [11]. Zhang D. et al. proposed a transformer maintenance decision-making approach based on comprehensive condition assessment. This approach achieved refined equipment health status assessment and priority ranking by introducing fuzzy mathematical methods or multi-attribute group decision-making [12]. Quiñones LIA et al. effectively identified high-risk transformers based on machine learning prediction results. They designed five types of maintenance activities for high-risk distribution transformers, and dynamically assigned tasks based on the specific risk characteristics of the transformers. This article verified the proposed maintenance strategy through real data [13]. M. Dong et al. explored a decision model based on reliability and economic evaluation for power transformers. The model can be used to determine the optimal time and type of maintenance for transformers. They proposed that in practical state maintenance strategies, a comprehensive balance should be made between failure rate, remaining lifespan, maintenance costs, and power outage losses [14]. Syah Afrizka Mareta Arum et al. proposed a maintenance strategy decision model for power transformers. The model combined health index (HI), statistical analysis, and life cycle cost (LCC), aiming to optimize the reliability and economy of maintenance strategies. Their model can identify specific faulty components based on health indices, perform local repairs or replacements, and provide updated transformer overhaul times [15].

Table 1 summarizes the above research. It can be found that the object hierarchy of these studies includes transformer groups, individual transformers, and components. That is, the maintenance strategies they studied were centered around the entire entity. This may bring a problem. Due to the complex composition of the transformer as a whole or its components, it is difficult to ensure that there are no omissions during maintenance. If maintenance strategies are determined based on specific failure modes, comprehensive maintenance of all failure can be achieved to reduce omissions. In addition, research specifically targeting failure modes can achieve more accurate identification and monitoring, thereby guiding personnel in selecting testing methods and maintenance actions. Especially for some failure modes that are difficult to identify or detect, it is necessary to carry out ‘customized’ maintenance. However, such a ‘customized’ effect is difficult to achieve in previous maintenance strategies.

**Table 1:** Research on transformer maintenance strategy.

Research	Research object hierarchy	Results of maintenance strategy obtained	Whether to consider the economic benefits?
Previous research [11]	Transformer group	Transformer maintenance sequence	No
Previous research [12]	Transformer group	Transformer maintenance sorting	Yes
Previous research [13]	Transformer group	Identify transformers that require maintenance and allocate maintenance task	Yes
Previous research [14]	Transformer	The optimal time and type for transformer maintenance	Yes
Previous research [15]	Transformer and component	Overhaul time for transformer and replacement plan for key components	Yes
This research	Specific failure modes of transformer components	Maintenance interval and failure modes risk level	Yes

The maintenance strategy results obtained from previous studies mainly included four categories: maintenance object positioning, maintenance sequence determination, maintenance type selection, and overhaul time update. Except for overhaul time being a quantifiable indicator, other results were not detailed enough. Furthermore, it is worth noting that the vast majority of studies considered economic benefits as an important decision-making factor, which provided important references for this article.

### 2.2. Risk analysis

Risk analysis, as an important research direction in decision science, engineering management, and public safety, aims to systematically identify, evaluate, and respond to potential adverse effects caused by various uncertain factors [16]. Common methods include Bayesian Analysis, FMECA, etc. [17].

Bayesian Analysis, as an important statistical inference method, has received widespread attention and application in various fields such as scientific research, engineering practice, and data applications in recent years [18]. Petar Sarajcev et al. proposed a preventive maintenance optimization method for power transformers based on Bayesian statistical learning and influence graphs. They attempted to solve the uncertainty problem in health status assessment and maintenance decision-making [11]. D. Paul et al. conducted fault diagnosis research on oil immersed transformers and reactors. They used dissolved gas analysis (DGA) data to identify and locate potential faults of different types. They proposed and implemented a Bayesian optimization based gradient boosting algorithm (BO-GB) for fault identification [19]. S. Li et al. comprehensively evaluated the health status of transformers on the basis of multi-source transformer condition monitoring data, and estimated the apparent age based on this. They proposed using Bayesian belief network (BBN) to implement probabilistic health index (PHI), and transformed the PHI into apparent lifespan through regression analysis. Their research provided reference for power companies in transformer operation, maintenance, and investment decision-making [20]. The sparsity, multivariate correlation, and abundant uncertain information of transformer failure data make Bayesian methods highly applicable in such problems. Bayesian methods provide more flexible analysis tools for operation and maintenance strategies, risk assessment, and life prediction.

Failure mode, effects and criticality analysis (FMECA) is a commonly used tool for conducting reliability analyses of equipment or systems. The risk prioritization number (RPN) method in FMECA is specifically designed to assess the risk level of failure modes. In general, when the risk priority number method is used, three influencing factors are considered: the occurrence probability ranking (OPR), effect severity ranking (ESR), and detection difficulty ranking (DDR) [21]. There may be several issues with this. Firstly, the weights assigned to the three risk factors are equal, which is clearly inconsistent with the reality [22]. Secondly, the assessment of each risk factor by risk assessors may be vague. Traditional methods have not considered using fuzzy theory to solve it [23]. Finally, considering only three risk factors is clearly not enough. Other risk factors should also be included in the scope of consideration on the basis of specific needs [24].

Many researchers have conducted improvement studies on RPN to address the above issues. Sorooshian, S improved the classic risk priority number method by assigning weights to three influencing factors for improving manufacturing systems [25]. Li, XX et al. used the risk priority number method to evaluate the biosafety of hospitals. They reestablished new influencing factors on the basis of research needs [26]. Romero Zayas et al. used a risk priority number method to quantitatively analyze the implementation risks of hospital radiology departments. They developed improvement measures for risk items. The method considered severity, probability, and detectability [27]. Wang, ZC et al. used an extended matter element model and AHP to determine the weights of risk influencing factors. The model determined the order of failure modes sorted by risk level. They verified the effectiveness of the method through its application on marine fishing vessels [28].

Previously, many scholars also have attempted to apply RPN to transformer related research. S. C. Freitag, Docki Saraswati, and Jaspreet Singh et al. applied traditional RPN methods to transformer failure research. They evaluated the risk level of failure modes, and attempted to further expand the RPN results [29–31]. S. C. Freitag established a new failure rate model based on RPN results to determine equipment failure status [29]. Docki Saraswati proposed three levels of maintenance urgency based on RPN results for fuzzy judgement of equipment maintenance needs [30]. Their researches were application studies of traditional RPN methods. But they cannot avoid the limitations brought by the original methods. Their application only provided vague results, difficult to guide the optimization of transformer maintenance strategies in practice. O. H. Eyüboğlu, A. P. Shafei, and Bin Zhou et al. improved the RPN formula. In some studies, risk factor weights were also considered [32–34]. Bin Zhou also considered using relevant methods to improve the subjective degree of weights [34]. Unfortunately, their research only stayed at the theoretical level and did not further expand the application of RPN. In addition, it has been noted that in previous studies, the specific analysis of RPN did not focus on the characteristics of transformer failure data or provide calculation methods suitable for small sample data. There is also a lack of benefit proof when applying or improving the methods. The specific summary is shown in the Table 2 below.

**Table 2:** Research on RPN in the field of transformers.

Research	Whether to improve the traditional calculation formula of RPN?	Whether to assign weights?	Whether to improve the subjective degree of weight?	Further applications of RPN?	Small sample failure calculation?	Proof of benefit?
Previous research [29]	No	No	No	Yes (A new failure rate model)	No	No
Previous research [30]	No	No	No	Yes (Three levels of maintenance urgency)	No	No
Previous research [31]	No	No	No	No	No	No
Previous research [32]	Yes (Added Age)	No	No	No	No	No
Previous research [33]	Yes (Added Age)	Yes	No	No	No	No
Previous research [34]	Yes	Yes	Yes	No	No	No
This research	Yes	Yes	Yes	Yes	Yes	Yes

### 2.3. Synthesis

On the basis of the above content, many reliability analyses and maintenance strategy formulation methods have been carried out, with a focus on transformers. These studies focused mostly on the overall transformer as the research object and rarely on plan maintenance from the perspective of failure modes. Moreover, cost factors affect maintenance strategies. That is, operation and maintenance costs significantly affect the formulation of transformer operation and maintenance strategies. In addition, as a commonly used risk assessment method, the RPN method can be used to conduct risk analyses of failure modes. But research in the field of transformers is very limited. This research extends upon the RPN method for transformers. When the RPN method is used, improvements and optimizations are often made from two perspectives: the selection of influencing factors and the calculation of their weights. Considering the importance of operation and maintenance costs, these factors are independently considered and assigned weights through the fuzzy AHP.

## 3. Materials and methods

**Fig 1:**
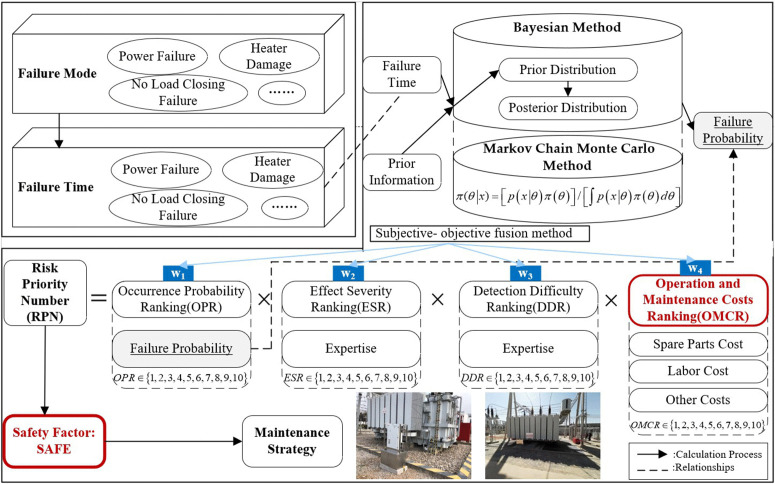
Method structure-calculate maintenance interval based on Bayesian and RPN.

To overcome the limitations of previous research, a reliability analysis and maintenance decision-making method for transformers based on failure modes is proposed. The core of this method is to refine the research focus to the failure mode of equipment, explore the reliability indicators related to failure probability, and utilize the powerful ability of Bayesian theory to integrate and optimize information from different sources, such as transformer operation data, maintenance records, and expert experience. The Bayesian and Markov chain Monte Carlo methods are used to calculate the probability of each failure mode and identify critical failure modes. By combining the failure probability threshold with the inverse mapping method, the benchmark operating time is obtained. Apply the RPN method that independently considers cost factors and assigns weights to obtain safety factors. On this basis, the fusion calculation results are used to optimize the maintenance interval. The framework of this research is illustrated in the following Fig 1.

### 3.1. Risk assessment and maintenance interval calculation

#### 3.1.1. Bayesian method and Markov Chain Monte Carlo method.

The collection of failure time data for transformers, as highly reliable large-scale power devices with complex working environments, is generally challenging, with insufficient samples in most cases. In small-sample scenarios, the failure probability can be calculated using the Bayesian method.

The Bayesian method is a statistical inference method based on the Bayesian theorem, which updates probabilities on the basis of prior probabilities and new data to obtain posterior probabilities. Firstly, it is necessary to establish a probability statistical model for the failure time of the equipment, that is, to determine the likelihood function. For failure time data, reliability models such as Weibull distribution, exponential distribution, or log normal distribution are often used to describe the life characteristics of equipment. Determining the appropriate prior distribution is a key step in Bayesian statistics. The prior distribution has a significant effect on the shape and range of the posterior distribution. Typically, it is necessary to integrate information from previous research, expert opinions, and relevant data, combined with professional knowledge, historical data, and statistical principles, to determine the prior distribution. The selection of priors can be non-informative priors, conjugate priors, or informative priors set based on expert experience.

The core of Bayesian inference is to combine the prior distribution with the likelihood function of the current data based on Bayes’ theorem to obtain the posterior distribution of parameters. In practical applications, the posterior distribution may be very complex and difficult to directly calculate or analytically solve. In a wide range of model spaces, the use of Markov chain Monte Carlo (MCMC) methods to sample the model space is effective for identifying models with high posterior probabilities [35–37].

The MCMC method is a statistical method based on random sampling that is suitable for estimating the parameters of complex probability distributions in situations in which analytical solutions are difficult to obtain. This method combines the properties of Markov chains and the sampling technique of Monte Carlo methods, and the probability distribution of equipment life targets is approximated on the basis of a random walk process. The core idea of the MCMC method is to construct a Markov chain with a stationary distribution that is the same as the target probability distribution [38]. By simulating this chain for a sufficiently long time, a sequence of states is generated, and the distribution of these states gradually approaches the target distribution. The samples extracted from this sequence can be used for statistical inference and reliability calculations, such as equipment failure probability calculations.

Common MCMC algorithms include Metropolis Hastings algorithm and Gibbs sampling, which define how to generate and accept new parameter states. It is necessary to ensure that the Markov chain has reached and stabilized at its steady distribution. This can be achieved by running multiple independent chains and observing their mixing, or by examining sample path diagrams, autocorrelation functions, etc. After confirming the convergence of the chain, the obtained samples can be regarded as independent samples from the posterior distribution. Based on these samples, the marginal posterior distribution, posterior mean, and confidence interval of parameters can be calculated, and ultimately the estimation of key reliability indicators such as equipment failure probability and reliability can be completed.

The MCMC method, as a flexible sampling method, can handle high dimensional and complex probability distributions in parameter space without adjusting calculation rules. It has the advantages of a wide application range and strong practicality and is the preferred spatial sampling method for Bayesian model averaging.

#### 3.1.2. Risk priority number method for independently considering costs.

Calculate the probability of failure based on the posterior distribution calculated in the previous section Taking the two parameter Weibull distribution as an example. The probability distribution function based on the Weibull distribution is as follows:


$$F_{failure\;\text{mod}e}=\text{1}-e^{-{\left(\frac{t}{\lambda_{weibull}}\right)}^{\beta }},t\geq \text{0}$$ (1)

A safety factor is introduced considering the complex effects of failure modes and difficulties in detection that may be encountered in practical engineering applications. The factor provides additional protection and avoids potential application risks associated with simple numerical calculations. In this study, the RPN method is used to determine the safety factor.

RPN is an indicator used in FMEA to quantify risk [39]. Notably, the risk level for a given failure is calculated on the basis of three factors: OPR, ESR, and DDR. Moreover, the risk of failure modes can be evaluated, maintenance resources can be reasonably allocated, and the overall reliability and safety of the equipment can be improved on the basis of the RPN calculation results. The corresponding formula is as follows:


RPN=OPR×ESR×DDR (2)

Considering the actual operation and maintenance work of transformers, separate analyses of operation and maintenance costs are innovatively incorporated into the RPN method on the basis of historical operation and maintenance cost data. In the original method, operation and maintenance costs were considered factors affecting the severity rating criteria. In this case, the impact of operational costs on failure modes is significantly underestimated. In this research, the RPN method is used in maintenance decision-making, and operation and maintenance costs, as decisive factors in maintenance decision-making, are given sufficient attention. Therefore, an operation and maintenance cost ranking (OMCR) variable is added to the RPN method. The modified RPN formula is as follows.


RPN=OPR×ESR×DDR×OMCR (3)

Notably, in the traditional RPN method, the same weight is assumed for several influencing factors, which is obviously unreasonable. In practical engineering, the sensitivity of different equipment or failure modes to influencing factors varies. When calculating the risk priority number, appropriate weights should also be assigned to different influencing factors. To solve the problem of weight allocation, the AHP is used to analyze four influencing factors. The AHP, a common mathematical method used for decision analysis, has the advantages of flexibility and strong adaptability and can be applied to various types of decision problems. To overcome the subjectivity and uncertainty of expert judgement in the traditional AHP and improve the robustness of weight calculation, the fuzzy AHP is applied, fuzzy logic is introduced, and the judgement matrix is modelled via the fuzzy AHP [40]. In the method, experts use triangular fuzzy numbers to express the relative importance of pairwise comparison criteria. Each element in the fuzzy judgement matrix is a three-parameter triangular fuzzy number, that is (l, m, u). The three parameters correspond to the lower limit, most likely value, and upper limit of the possible interval for judgement. Firstly, calculate the fuzzy geometric mean of each criterion’s corresponding row, and then normalize it to obtain the fuzzy weights. Finally, the fuzzy weights are deblurred by the centroid method. This method effectively reflects the ambiguity of expert judgement and reduces the single point estimation error of judgement.

Based on the obtained fuzzy weights, a method of integrating subjective and objective weights is adopted to further reduce the risks that subjective judgments may bring. Objective weights are obtained through three methods: CRITIC, Entropy, and StdDev. These three methods are common objective weight determination methods. These methods are entirely based on the statistical characteristics of the data itself to determine criterion weights, without relying on the subjective judgments and preferences of decision-makers. When using the three methods to obtain results, different weights will be assigned to each result. The CRITIC method is a comprehensive analytical approach that considers both the strength of data comparison and the correlation between criteria. Therefore, give this result a higher weight. The Entropy method has a theoretical foundation based on information theory and advantages in information quantification. But this method ignores correlation, thus obtaining moderate weight. The StdDev method is computationally simple but has limited information content. And this method completely ignores the correlation between indicators. Therefore, this method obtains the lowest weight.

For transformers, due to insufficient samples of certain low-frequency but high-risk failure modes in historical data, purely data-driven methods may not be able to fully identify these key risk factors. And expert judgment can supplement the incompleteness of data based on theoretical knowledge and practical experience. Therefore, in the process of weight fusion, fuzzy weight results are still the main focus.

The final weight determination formula used in this study is as follows:


$$w_{i}=0.8\times w_{AHP}+0.2\times \left(0.5\times w_{CRITIC}+0.3\times w_{Entropy}+0.2\times w_{StdDev}\right),i=1,2,3,4$$ (4)

Among them, w_i_ is the weight of the i-th influencing factor, and w_AHP_, w_CRITIC_, w_Entropy_, and w_StdDev_ are the weight results obtained through four methods, respectively.

The formula for the RPN method after weight analysis is as follows:


$$RPN=\left(w_{1}OPR\right)\times \left(w_{2}ESR\right)\times \left(w_{3}DDR\right)\times \left(w_{4}OMCR\right)$$ (5)

Where w_1_, w_2_, w_3_, and w_4_ are the weights corresponding to the relevant influential factors.

A specific introduction to each influencing factor is provided below. The OMCR is used to evaluate the operation and maintenance costs associated with a certain failure mode of a transformer. Operation and maintenance costs generally include spare part costs, labor costs, and other costs caused by failures. The lower the operation and maintenance costs are, the lower the failure mode score. Table 3 shows the scoring criteria for the OMCR.

**Table 3:** OMCR criteria.

OMCR level	Operation and maintenance cost level	Operation and maintenance cost C_m_ reference range (RMB)
1-2	Very low	$0<C_{m}\leq 30000$
3-4	Low	$30000<C_{m}\leq 50000$
5-6	Medium	$50000<C_{m}\leq 100000$
7-8	High	$100000<C_{m}\leq 200000$
9-10	Very high	$C_{m}>200000$

The OPR is used to estimate the likelihood of a specific failure mode occurring in a transformer. Table 4 shows the rating criteria for OPR, where the ‘probability of failure mode occurrence P_m_ reference range’ represents the rating level corresponding to the expected probability of failure mode occurrence throughout the entire service life of the transformer. The lower the likelihood of failure mode occurrence is, the lower the score.

**Table 4:** OPR criteria.

OPR level	The likelihood of failure occurring	Probability of failure mode occurrence P_m_ reference range
1	Extremely low	$P_{m}\leq 10^{-6}$
2-3	Low	$1\times 10^{-6}<P_{m}\leq 1\times 10^{-4}$
4-6	Medium	$1\times 10^{-4}<P_{m}\leq 1\times 10^{-2}$
7-8	High	$1\times 10^{-2}<P_{m}\leq 1\times 10^{-1}$
9-10	Very high	$P_{m}>10^{-1}$

The ESR is used to assess the severity of the potential impact of a specific failure mode. The milder the impact is, the lower the failure mode score. Table 5 shows the ESR criteria.

**Table 5:** ESR criteria.

ESR level	Severity level	The severity of the impact of failure
1-3	Light	Not enough to cause personal injury, minor damage to the transformer, and minor environmental damage, but it can lead to unplanned maintenance or repair of the transformer
4-6	Medium	Causes moderate injuries to personnel, moderate damage to transformers, task delays or downgrades, and moderate environmental damage
7-8	Fatal	Causes serious injury to personnel, severe damage to transformers, task failure, and serious environmental damage
9-10	Catastrophic	Causes death of personnel, damage to transformers, and significant environmental damage

The DDR is used to assess the degree of difficulty with which a specific failure mode can be detected during maintenance work. The more easily detectable the failure mode is, the lower the score. Table 6 shows the scoring criteria for the DDR.

**Table 6:** DDR criteria.

DDR level	Detection difficulty level	The difficulty level of failure detection
1-2	Highly detectable	The failure modes are very easy to identify and detect, and failures can be immediately detected using standard detection methods or during normal operation
3-5	Easy to detect	Failure modes are relatively easy to detect and may require some basic testing or inspection processes, but they usually do not require highly specialized tools or knowledge
6-8	Difficult to detect	Failure modes are difficult to detect and may require special testing equipment, detailed diagnostic procedures, or specific technical knowledge
9-10	Extremely difficult to detect	Failure modes are extremely difficult to detect and may not be accurately identified even with advanced testing and diagnostic methods

**Fig 2:**
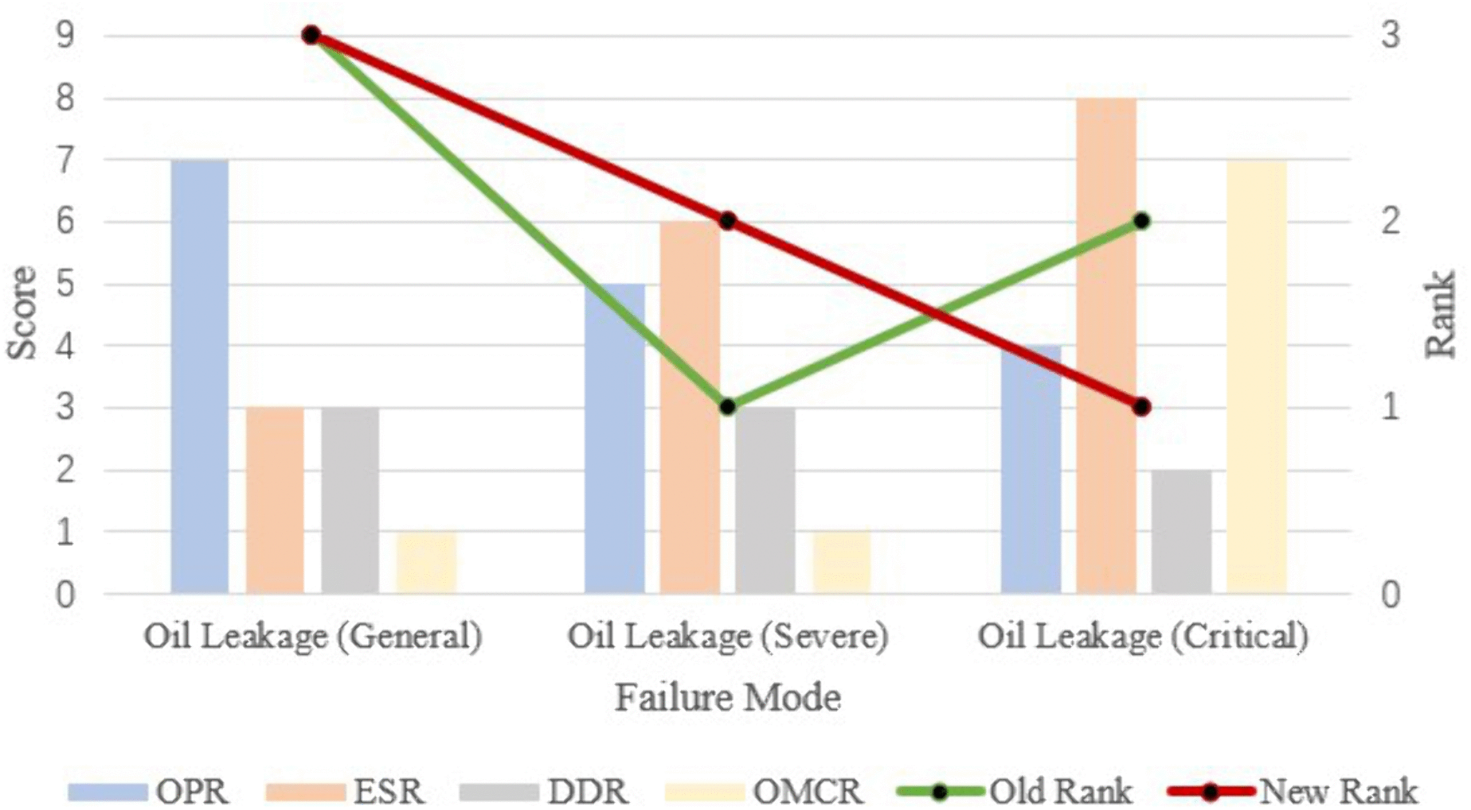
Risk ranking example.

With oil leakage as an example, the effectiveness of the new formula is demonstrated. First, we score and assign weights to the risk factors for each failure mode, as shown in Table 7. In the old method, the result calculated on the basis of three risk factors was that oil leakage (severe) was classified at the highest risk and that oil leakage (general) was classified at the lowest risk. With the new method, the risk ranking changed. Oil leakage (critical) is classified as the failure mode with the highest risk because of its significant impact on cost. The ranking is shown in Fig 2. Therefore, for cost-oriented failure mode assessment, treating cost independently as a risk factor can effectively increase the consideration of cost.

**Table 7:** Example rating.

Component name	Failure mode	OPR (w_1_ = 0.2077)	ESR (w_2_ = 0.0609)	DDR (w_3_ = 0.0474)	OMCR (w_4_ = 0.6839)	Failure description
Oil storage tank	Oil leakage (general)	7	3	3	1	The oil leakage rate should not be faster than 5 seconds per drop, and the oil level should be normal.
Oil storage tank	Oil leakage (severe)	5	6	3	1	The oil leakage rate is faster than 5 seconds per drop, and the oil level is normal.
Oil storage tank	Oil leakage (critical)	4	8	2	7	Oil leak forms an oil flow, the oil leakage rate is faster than 5 seconds per drop, or the oil level is below the lower limit.

### 3.2. Development of maintenance strategy

On the basis of the RPN, the safety factor was determined according to the following principles:

4) Under the same conditions, the higher the probability of failure is, the lower the safety factor of the failure mode.
5) Under the same conditions, the higher the severity level is, the lower the safety factor of the failure mode.
6) Under the same conditions, the higher the difficulty of detection is, the lower the safety factor of the failure mode.
7) Under the same conditions, the higher the total operation and maintenance cost is, the lower the safety factor of the failure mode.

According to the above principles, the magnitude of the safety factor is inversely proportional to the RPN, that is, the failure mode with the lowest safety factor should be considered first. Notably, the safety factor SAFE can be calculated via the following formula:


$$SAFE=\text{1}-\left[\frac{w_{\text{1}}OPR}{\text{sup}(G)}+\frac{w_{\text{2}}ESR}{\text{sup}(G)}+\frac{w_{\text{3}}DDR}{\text{sup}(G)}+\frac{w_{\text{4}}OMCR}{\text{sup}(G)}\right],\;G=\left\{\text{1},\text{2},\text{3},\text{4},\text{5},\text{6},\text{7},\text{8},\text{9},\text{1}\text{0}\right\}$$ (6)

The safety factor SAFE is a core parameter that connects the risk characteristics of failure modes with the configuration of maintenance strategies. Firstly, the safety factor is used to correct the inefficiency. The calculation formula is shown in Formula 7, where k is the risk amplification factor. The determination of k value follows the principle of reverse response of SAFE. Extremely high risk: k = 2.5–2.8, indicating that the actual risk far exceeds the prediction of Weibull theory. High risk: k = 2.0–2.2. Medium risk: k = 1.6–1.8. Low risk: k = 1.5–1.6, moderate correction intensity. This adaptive mechanism ensures comparability of cost models for different risk levels and failure modes, while maintaining sensitivity to actual risks.


$$\lambda_{adjusted}=\lambda \times \left[1+k\times \left(1-SAFE\right)\right]$$ (7)

Secondly, the safety factor is used for weight allocation of various maintenance strategies. The maintenance strategy proposed in this study is a comprehensive strategy that integrates four basic maintenance strategies. Includes emergency response, predictive maintenance, condition monitoring, and scheduled maintenance. The weights of the four strategies are calculated using the following formulas:


$$w_{emergency}=\alpha_{1}\times {\left(1-SAFE\right)}^{2}$$ (8)

$$w_{predictive}=\alpha_{2}\times \left(1-SAFE\right)$$ (9)

$$w_{condition}=\alpha_{3}\times SAFE$$ (10)

$$w_{time}=\alpha_{4}\times SAFE^{2}$$ (11)

$$w_{total}=w_{emergency}+w_{predictive}+w_{condition}+w_{time}$$ (12)

$$w_{emergency\_final}=w_{emergency}/w_{total}$$ (13)

$$w_{predictive\_final}=w_{predictive}/w_{total}$$ (14)

$$w_{condition\_final}=w_{condition}/w_{total}$$ (15)

$$w_{time\_final}=w_{time}/w_{total}$$ (16)

Among them, a₁, a₂, a₃, and a₄ are the benchmark weight coefficients set according to the characteristics of the failure mode. After the weight allocation is completed, the abstract weight percentage needs to be converted into specific maintenance actions and cost expenditures through a cost conversion mechanism. This conversion process involves three core steps: determining the annual maintenance frequency, weighted calculation of single maintenance costs, and dynamic accumulation of total costs.

The total frequency of annual maintenance is not a fixed value, but is dynamically determined by the SAFE value. The calculation process is shown in Formula 17:


$$M_{annual}=M_{base}+\left(1-SAFE\right)\times RISK_{multiplier}$$ (17)

Among them, M_annual_ is the total annual maintenance frequency, M_base_ is the basic maintenance frequency, and RISK_multiplier_ is the risk response multiplier. If the risk response multiple is 6, it means that for every 10% increase in risk, there will be an increase of 0.6 annual repairs.

The actual cost of each repair is the result of a weighted mixture of the costs corresponding to the four types of repairs. The annual maintenance frequency and single maintenance cost will directly affect the total cost. The calculation process is shown in Formula 18:


$$C_{per\_maintenance}=w_{emergency}\times C_{emergency}+w_{predictive}\times C_{predictive}+w_{condition}\times C_{condition}+w_{time}\times C_{time}$$ (18)

The total cost consists of preventive maintenance costs, corrective maintenance costs, downtime loss costs, and monitoring costs. The calculation formula is as follows:


$$C\left(t\right)=C_{preventive}\left(t\right)+C_{corrective}\left(t\right)+C_{downtime}\left(t\right)+C_{monitoring}\left(t\right)$$ (19)

$$C_{preventive}\left(t\right)=C_{per\_maintenance}\times M_{annual}$$ (20)

$$C_{corrective}\left(t\right)=C_{emergency}\times \left[\lambda_{adjusted}\times \left(1-E_{prevention}\right)\left(1+F_{aging}\times t\right)\right]\times 8760$$ (21)

$$C_{downtime}\left(t\right)=R_{downtime\_\cos t}\times MTTR\times M_{annual}$$ (22)

$$C_{monitoring}\left(t\right)=C_{monitoring\_base}\times \left[1-R_{learning}\times \ln\left(t+1\right)\right]$$ (23)

Among them, E_prevention_ is the failure prevention effect, and F_aging_ is the aging coefficient. R_downtime_cost_ is the downtime loss rate (yuan/hour), reflecting the importance of the equipment. MTTR is the average repair time (in hours), and C_monitoring_base_ is the initial monitoring cost. R_learning_ is the learning rate, which reflects the efficiency improvement brought by data accumulation.

In addition, a 20-year net present value NPV calculation has been introduced, with the following formula:


$$NPV=\sum_{t=1}^{20}\frac{C\left(t\right)\times {\left(1+i\right)}^{t-1}}{{\left(1+r\right)}^{t}}$$ (24)

Among them, i is the inflation rate and r is the discount rate.

## 4. Case study

In this research, the 220 kV oil-immersed transformers were selected as a case study to calculate failure probability and maintenance interval period. This type of transformer is a high-voltage power transformer mainly used for voltage conversion in transmission lines systems, that is, to reduce electrical energy from higher voltage levels to lower voltage levels for use in urban power grids and large industrial facilities. Owing to its high operating voltage, this type of transformer has special design and safety requirements.

The studied transformers are used across the entire Inner Mongolia Province. The collected transformer data are from two main sources: sensors mounted on transformers and onsite records from inspection personnel. Before conducting actual calculations, bad data, including erroneous data and fuzzy data, are removed. These bad data are generated mainly by manual recording errors.

First, on the basis of the data provided by the Inner Mongolia Power Company, the component names and corresponding equipment operation times and failure occurrence times for different failure modes are obtained.

The formula proposed earlier is used to calculate the difference between the failure occurrence time and the operation time to obtain the failure time data for different failure modes corresponding to different components. The failure times corresponding to these failure modes are summarized in Table 8.

**Table 8:** Failure occurrence time data.

Component name	Failure mode	Failure time (h)
Load tap changer	Power failure	14300, 17100, 22304, 35200, 46330, 46835, 56603, 60120, 60400, 82935, 90395, 110200, 113168, 120106, 132580, 136602, 140540, 143949, 151184,161100, 197025
	Heater damage	118959, 81188, 123353, 140182, 81802, 58391, 73425, 73143, 74416, 35314, 48429, 48188, 6092, 51498, 67964, 67594, 14889, 39186, 61952, 66154, 66154, 25857, 58553
	No-load closing	197169, 149461, 89291, 106187, 217809, 214161, 135975, 90251, 135567, 77076, 40455, 60731, 22363
Cooling system	Abnormal operation of the fan	185373, 98205, 40043, 2983, 46425, 1897, 178015, 230794, 70192, 50385, 52914, 8528, 101986, 6780, 20273, 33108, 10059, 1877, 58146, 51696
Gas relay	Damaged rainproof measures	125200, 139142, 117398, 117398, 147825, 95408, 117680, 92650, 74770
	Light gas transmission	58256, 228714, 2060, 142543, 115671, 96201, 39442, 5218, 46995, 1562, 1295, 48326, 2208, 3304, 17815, 27573, 35516
	Oil leakage	116897, 80408, 141801, 129573, 266722, 67521, 93490, 43048, 15130, 44799, 26289, 39995, 17216, 74920, 14004, 28954, 41169
Pressure relief valve	Oil seepage	24045, 29701, 1777, 19661, 5616, 11177, 2314, 8998, 3554, 8377, 16751, 32817, 19013, 37962, 2184, 45424, 32265, 17573, 7060, 4354, 24801, 3840, 5143, 1792, 22014, 4467, 44961, 9100, 9411, 40666, 47008, 16744, 20375, 1905, 6993
Bushing	Heating	258849, 261513, 147920, 251626, 63452, 38901, 43907, 78284, 87164, 34440, 24685, 150213, 15138, 146217, 140056, 16541, 32159, 223052, 16056, 54764, 107483, 92459, 26073, 33313, 185587, 4393, 155566, 105441, 86194, 113610, 87089, 119075, 64288, 3699, 5737, 90012, 88952, 85068, 127917, 8086, 77731, 59877, 83710, 9551, 84982, 79963, 5428, 43724, 16904, 1128, 50240, 39056, 13163, 2976, 67957, 89315, 74363, 47028, 8194, 50697, 3168, 34556

The lifespan of transformers usually follows the Weibull distribution, and therefore, the Weibull distribution is chosen as the preferred distribution. The Weibull distribution is a continuous probability distribution widely used in fields such as engineering, meteorology, and insurance, especially in reliability and survival analysis [41]. The Swedish mathematician Wald Weibull provided a detailed explanation of the Weibull distribution in 1951. The Weibull distribution is commonly used to describe phenomena such as material fatigue life and equipment failure [42–44]. The shape of the Weibull distribution is very flexible, and it can be used to simulate different types of data distributions by changing its shape and scale parameters. The shape parameter influences the shape of the distribution, whereas the scale parameter influences the scale or range of the distribution.

Next, the Bayesian and MCMC methods are used to calculate the failure probabilities corresponding to some typical failure modes. The specific verification process is as follows.

After determining the Weibull distribution as the likelihood function, the next step is to determine the prior distribution. Select prior distributions by combining domain knowledge and data-driven validation. Firstly, based on the engineering experience of transformer equipment and the physical meaning of Weibull distribution parameters, three different types of prior information were designed. They are information prior, weak information prior, and no information prior. Then generate prediction samples through prior prediction checks. Finally, by comparing the impact of different prior types on posterior inference, the most suitable prior distribution for the current data and analysis objectives is selected.

**Fig 3:**
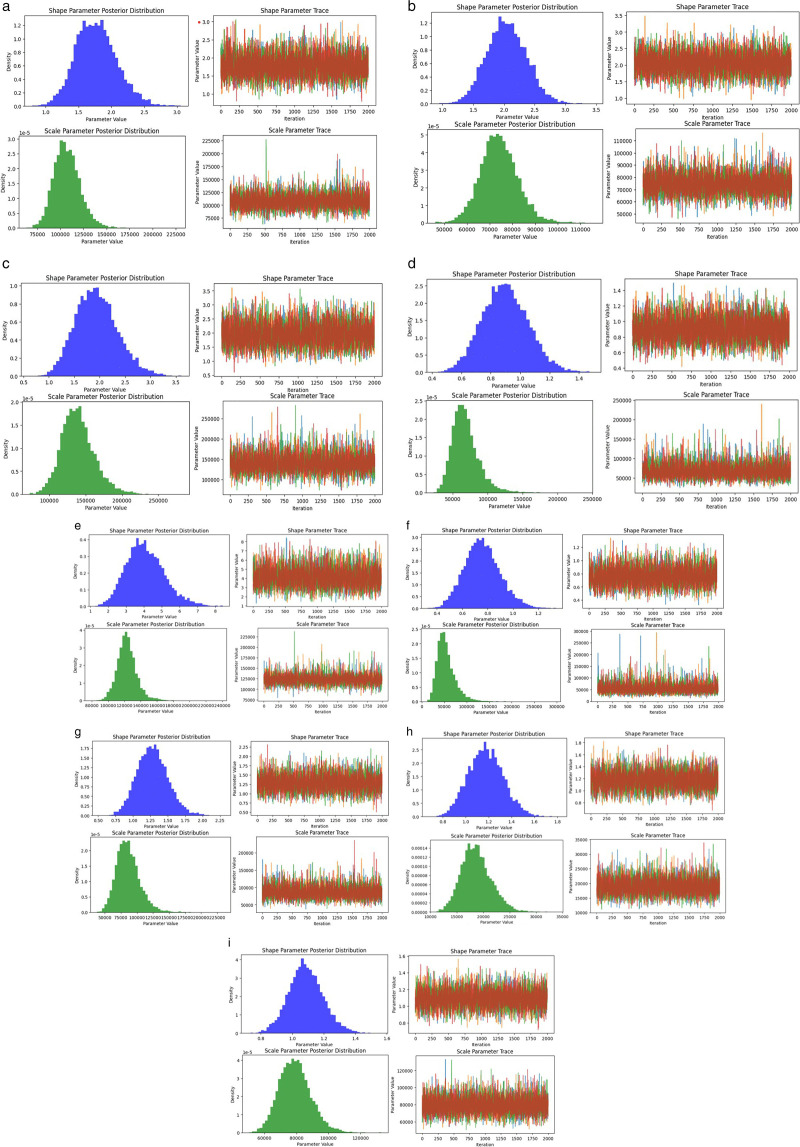
MCMC trace diagram. (a) power failure; (b) heater damage; (c) no-load closing failure; (d) abnormal operation of the fan; (e) damaged rainproof measures; (f) light gas transmission; (g) oil leakage; (h) oil seepage; (i) heating.

The graphs in Fig 3 visualize the posterior sampling results of key parameters in the Bayesian model. The left side of the figure shows the posterior distribution of the shape and scale parameters, demonstrating the distribution characteristics of the converged sampling chain with normal shapes. On the right are the trace plots corresponding to the parameters, with different sampling chains represented by curves with different shapes, which are used to assess the stability and effects of parameter values during the sampling process. The horizontal axis represents the number of sampling steps, and the vertical axis represents the parameter values. The graph shows that the posterior distributions of multiple samples are highly consistent and that the parameter trajectories oscillate around a stable center throughout the entire sampling interval, without obvious trend drift or abnormal jumps. This indicates that the MCMC sampling process fully converges and the collected samples can be used to effectively characterize the posterior distribution of the target.

**Fig 4:**
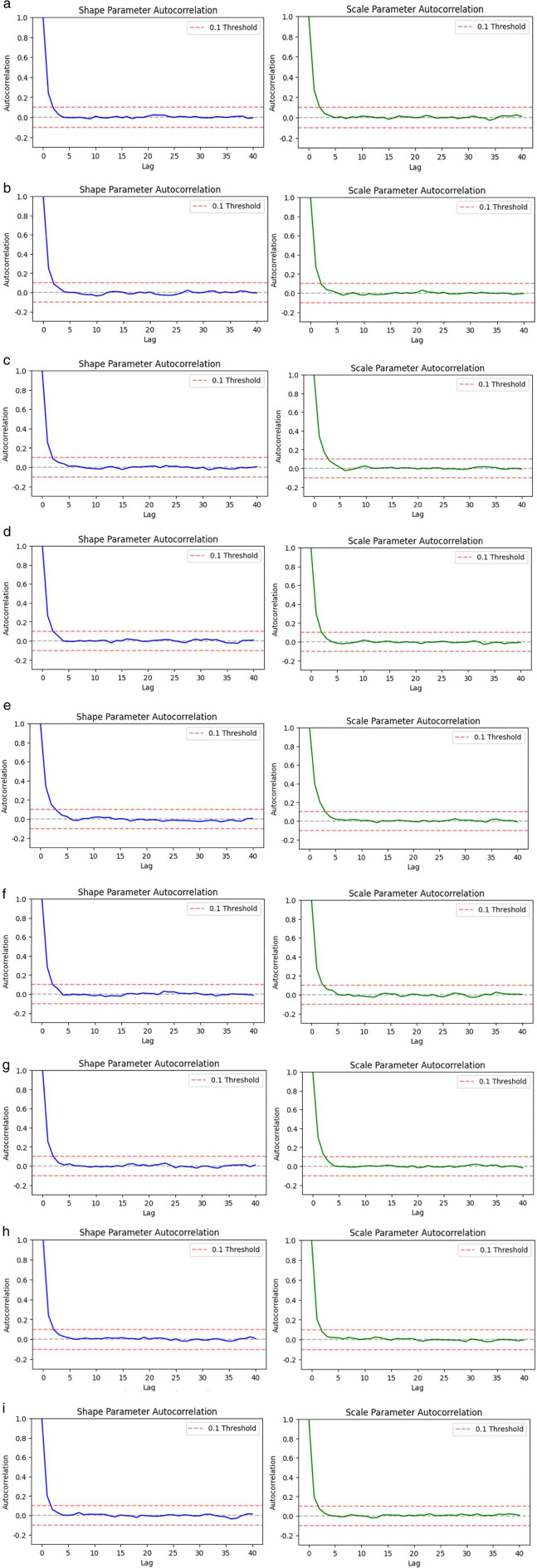
MCMC autocorrelation diagram. (a) power failure; (b) heater damage; (c) no-load closing failure; (d) abnormal operation of the fan; (e) damaged rainproof measures; (f) light gas transmission; (g) oil leakage; (h) oil seepage; (i) heating.

The graphs in Fig 4 demonstrate the autocorrelation structure of the parameters shape and scale during MCMC sampling, and the plots are used to evaluate the exploration efficiency and convergence of Markov chains in reference to the posterior distribution. In the figure, the autocorrelation functions of the shape parameter and scale parameter are shown separately. The horizontal axis represents the lag steps (lag), and the vertical axis represents the corresponding autocorrelation coefficient. The graph shows that the autocorrelation of all chains rapidly decays to near zero after a small lag step, and there is no significant autocorrelation for a long time. This indicates that there is strong independence and good mixing among the samples, additionally, the MCMC process can efficiently traverse the parameter space. The results demonstrate the effectiveness of the model sampling approach and the reliability of the posterior estimation.

The following Table 9 shows the specific parameters of the prior and posterior distributions:

**Table 9:** Specific parameters of the prior and posterior distributions.

Failure mode	Prior type	Prior distribution	Posterior distribution parameters
Power failure	informative	Shape ~ Gamma (alpha = 3.0, beta = 1.5)	Alpha = 5.00, Beta = 2.00
		Scale ~ Uniform (10000, 500000)	
Heater damage	informative	Shape ~ Gamma (alpha = 3.0, beta = 1.5)	Alpha = 3.00, Beta = 2.00
		Scale ~ Uniform (10000, 500000)	
No-load closing failure	informative	Shape ~ Gamma (alpha = 3.0, beta = 1.5)	Alpha = 5.00, Beta = 2.00
		Scale ~ Uniform (10000, 500000)	
Abnormal operation of the fan	informative	Shape ~ Gamma (alpha = 3.0, beta = 1.5)	Alpha = 3.00, Beta = 2.00
		Scale ~ Uniform (10000, 500000)	
Damaged rainproof measures	informative	Shape ~ Gamma (alpha = 3.0, beta = 1.5)	Alpha = 5.00, Beta = 1.25
		Scale ~ Uniform (10000, 500000)	
Light gas transmission	informative	Shape ~ Gamma (alpha = 3.0, beta = 1.5)	Alpha = 1.00, Beta = 1.25
		Scale ~ Uniform (10000, 500000)	
Oil leakage	informative	Shape ~ Gamma (alpha = 3.0, beta = 1.5)	Alpha = 1.00, Beta = 1.25
		Scale ~ Uniform (10000, 500000)	
Oil seepage	informative	Shape ~ Gamma (alpha = 3.0, beta = 1.5)	Alpha = 1.00, Beta = 2.00
		Scale ~ Uniform (10000, 500000)	
Heating	informative	Shape ~ Gamma (alpha = 3.0, beta = 1.5)	Alpha = 3.00, Beta = 2.00
		Scale ~ Uniform (10000, 500000)	

In addition to diagnostic charts, metrics such as the Gelman-Rubin diagnostic indicators are also calculated to assess convergence. The specific results are shown in Table 10. The convergence judgement parameters are all within a reasonable range, and the MCMC converges.

**Table 10:** MCMC convergence results.

Failure mode	Parameter	Posterior mean	Gelman-Rubin Statistic (R-hat ≤ 1.01)
Power failure	shape	1.853	1.0001
	scale	107478.4	1.0006
Heater damage	shape	1.982	1.0000
	scale	73477.2	1.0009
No-load closing failure	shape	2.036	1.0007
	scale	141877.6	1.0014
Abnormal operation of the fan	shape	0.895	1.0012
	scale	67988.3	1.0016
Damaged rainproof measures	shape	5.140	1.0005
	scale	123699.6	1.0008
Light gas transmission	shape	0.710	1.0003
	scale	53344.8	1.0004
Oil leakage	shape	1.238	1.0014
	scale	85427.8	1.0008
Oil seepage	shape	1.135	1.0006
	scale	18680.7	1.0004
Heating	shape	1.085	1.0022
	scale	79264.6	1.0009

**Fig 5:**
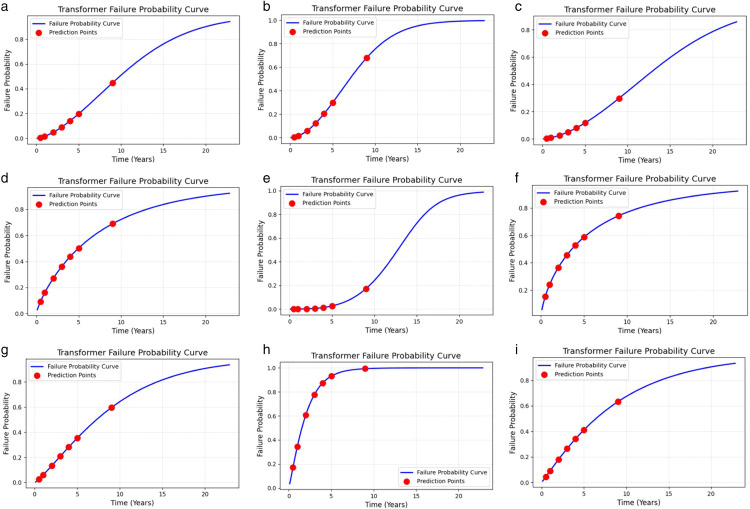
Failure probability prediction chart. (a) power failure; (b) heater damage; (c) no-load closing failure; (d) abnormal operation of the fan; (e) damaged rainproof measures; (f) light gas transmission; (g) oil leakage; (h) oil seepage; (i) heating.

The Fig 5 shows the posterior probability of device failure over time at different time points. With increasing time, the cumulative failure probability gradually increases, indicating that the longer the equipment is used, the greater the possibility of failure. Notably, the overhaul period of transformers is generally 9 years, so when the probability of failure is calculated, the input time is 78840 hours. The same time input ensures that each failure mode can be distinguished according to its probability of occurrence under the same standard. The results under these conditions are valid and acceptable. The probability of occurrence corresponding to 78840 hours calculated is shown in the following Table 11:

**Table 11:** Failure probability.

Failure mode	Failure probability	95% confidence interval of failure probability
Power failure	0.4475	[0.2754, 0.5994]
Heater damage	0.6794	[0.5318, 0.8303]
No-load closing failure	0.2975	[0.1224, 0.4948]
Abnormal operation of the fan	0.6885	[0.5272, 0.8433]
Damaged rainproof measures	0.1715	[0.0301, 0.3574]
Light gas transmission	0.7450	[0.5681, 0.9043]
Oil leakage	0.5975	[0.4033, 0.7695]
Oil seepage	0.9925	[0.9740, 1.0000]
Heating	0.6324	[0.5373, 0.7278]

The reasons for these results can be summarized as follows:

1) The heater is often one of the oldest electrical/thermal components inside an on-load switch. Owing to thermal expansion and contraction, mechanical fatigue, insulation ageing, and thermal corrosion, the fatigue life of materials is limited, especially when subjected to frequent heating, cooling, or overload, which exacerbates ageing.
2) The power supply section generally withstands large and varying current and voltage shocks but may have better protection and a slightly longer lifespan than the heater.
3) Switches are mainly responsible for opening and closing operations, and mechanical fatigue is the main failure mode; however, switches are not used as often in operation as other components are, so the failure rate is relatively low.
4) Oil seepage is the most likely failure mode to occur, which is very consistent with the actual situation. The transformer oil seal system is most prone to aging during long-term operation, and the deterioration of seals leading to oil leakage is the most common problem.
5) The probability of light gas transmission is relatively high. Because it is sensitive to various abnormalities inside the transformer, including slight partial discharge, overheating, etc.
6) Cooling fans are mechanical moving parts, and problems such as bearing wear and motor aging are common after long-term operation, with a high probability of failure.
7) During long-term operation, transformers have a higher probability of local overheating due to factors such as load changes, decreased cooling system efficiency, and insulation aging.
8) The probability of oil leakage is lower than that of oil seepage, but still higher. Oil leakage is usually the result of further deterioration of oil seepage issues.
9) The rainproof facilities of outdoor transformers may indeed be damaged under harsh weather and long-term exposure, but the probability is relatively low because these facilities are usually designed to have a longer lifespan.

The weights of the factors that influence risk determined with the fuzzy AHP are shown in Table 12.

In Table 12, it can be noted that the CR value is 0.0518, which is less than 0.1, indicating that the matrix has an acceptable level of consistency. The subjective weights obtained are 0.2077, 0.0609, 0.0474, and 0.6839, respectively. Next, continue to calculate the objective weights. The decision matrix used for calculating objective weights is shown in the following Table 13:

**Table 12:** Weight table of influencing factors.

	OPR	ESR	DDR	OMCR	w_i_	CR
OPR	1.0000	5.0000	4.0000	0.3333	0.2077	0.0518
ESR	0.2000	1.0000	2.0000	0.1250	0.0609	
DDR	0.2500	0.5000	1.0000	0.1429	0.0474	
OMCR	3.0000	8.0000	7.0000	1.0000	0.6839	

**Table 13:** Decision matrix.

Array	Explanation
[8.2, 7.1, 7.0, 9.9]	High probability, high impact, difficult to detect, and costly to prevent failure modes
[7.5, 8.2, 6.8, 3.8]	Failure with high probability and high impact but relatively low cost
[6.8, 6.9, 8.1, 7.2]	Moderate probability and impact, but difficult and costly to detect
[9.1, 6.5, 7.3, 9.1]	High probability but moderate impact failure, with extremely high prevention costs
[7.2, 9.0, 6.9, 2.9]	Failure modes with high probability of serious consequences but low cost

This matrix is mainly used to make refined management decisions for identified high-risk failure modes. In the practice of transformer operation and maintenance, when equipment enters the aging period or warning signals appear, managers need to prioritize and allocate resources among multiple potentially serious failure. At this point, the comparative value of low-risk scenarios is limited, so choosing high-risk scenarios has more practical value. Calculate objective weights based on this matrix. The calculation results of all weights are shown in the following Table 14:

**Table 14:** Fusion weight table of influencing factors.

Influencing factor	Subjective weight (AHP)	Objective weight (CRITIC)	Objective weight (Entropy)	Objective weight (StdDev)	Comprehensive objective weight	Fusion weight
OPR	0.2077	0.2730	0.0483	0.25	0.2010	0.2064
ESR	0.0609	0.2095	0.0660	0.25	0.1745	0.0836
DDR	0.0474	0.2930	0.0186	0.25	0.2021	0.0783
OMCR	0.6839	0.2245	0.8671	0.25	0.4224	0.6316

**Fig 6:**
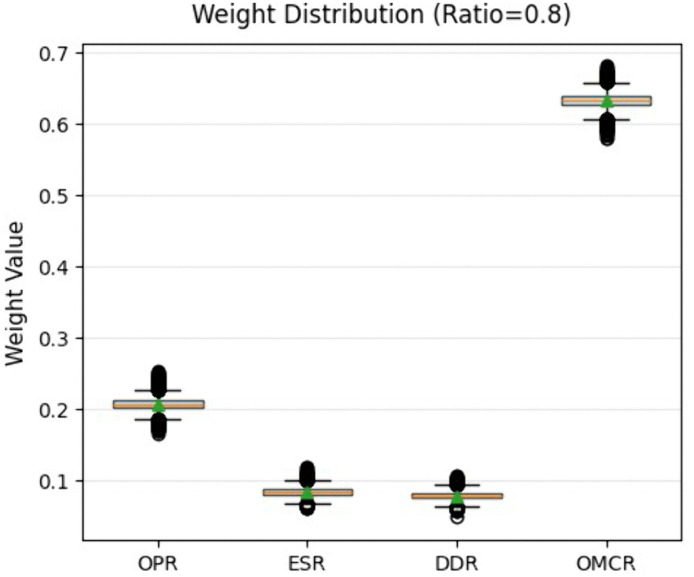
Weight distribution chart.

Perform sensitivity analysis on the above process using Monte Carlo simulation technology. Adopting a high concentration Dirichlet distribution for weight perturbation modeling and introducing a stability enhanced sampling mechanism. Partial simulations use original weights and small perturbations instead of completely random sampling. The sensitivity analysis results obtained are shown in Fig 6:

Fig 6 is based on the results of Monte Carlo simulation and is presented in the form of a box plot. This graph shows the probability distribution characteristics and robustness performance of the weights of each criterion. The analysis results show that the weight system exhibits a significant hierarchical structure. The weight of the OMCR criterion is concentrated in the range of [0.60, 0.67], with a compact box height and few outliers. This indicates that the dominant criterion has strong robustness to parameter perturbations. The weight distribution of the OPR criterion is in the range of [0.18, 0.25], with moderate interquartile range, showing good but slightly inferior stability. The weights of ESR and DDR criteria are both in the low range of [0.06, 0.12], and their relatively wide distribution range reflects the sensitivity of secondary criteria to input changes. From a statistical perspective, the minimal variance feature of the dominant criterion ensures the core logical stability of the decision-making framework. The moderate variability of secondary criteria reflects the model’s ability to respond reasonably to environmental uncertainty. This distribution feature validates the effective balance achieved by the fusion weight method between maintaining the stability of key elements and system adaptability, providing statistical support for the reliability of multi criteria decision-making.

**Fig 7:**
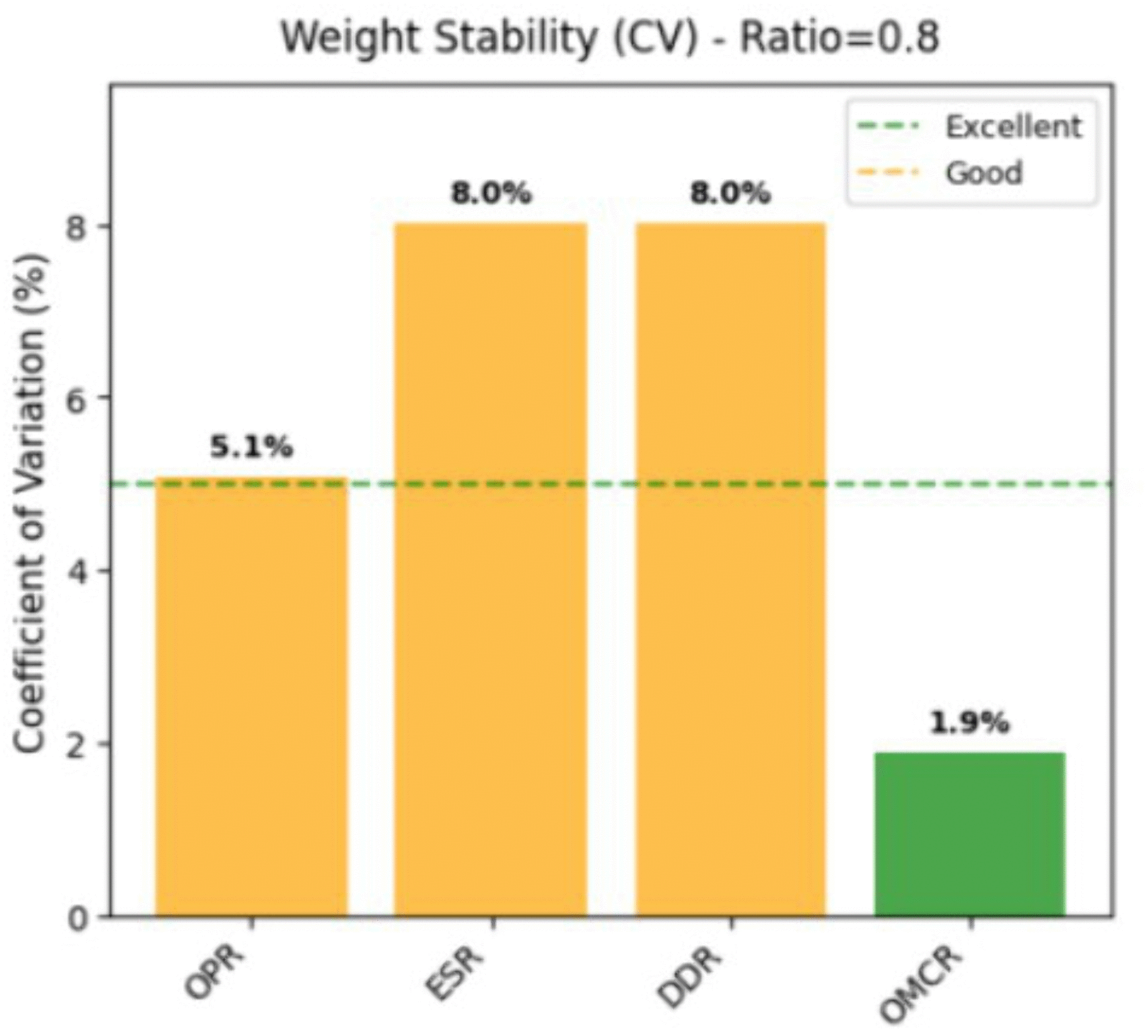
Weight stability chart.

Fig 7 quantitatively evaluates the relative stability level of each criterion weight through the coefficient of variation (CV), a dimensionless standardized indicator. The results indicate that the stability of the criteria exhibits a gradient distribution characteristic. The OMCR criterion has a CV value of 1.9%, which meets the excellent stability standard (CV < 5%) and demonstrates outstanding anti-interference characteristics. The CV value of the OPR criterion is 5.1%, which falls within the good stability range (5% ≤ CV < 10%) and demonstrates acceptable parameter sensitivity. The CV values of ESR and DDR criteria are both 8.0%, which is within a good stability range but close to the critical threshold. This result indicates that the fusion weight strategy has successfully achieved optimized configuration. By protecting the robustness of the dominant criteria to maintain consistency in decision-making logic, while allowing secondary criteria to adjust within a reasonable range to adapt to environmental changes. The quantitative analysis results not only verify the theoretical rationality of the subjective objective weight fusion method, but also provide empirical evidence for the robustness evaluation of the practical application of multi criteria decision support systems.

The collaborative analysis of Fig 6 and Fig 7 shows that the constructed fusion weight model exhibits excellent statistical robustness and decision reliability.

**Fig 8:**
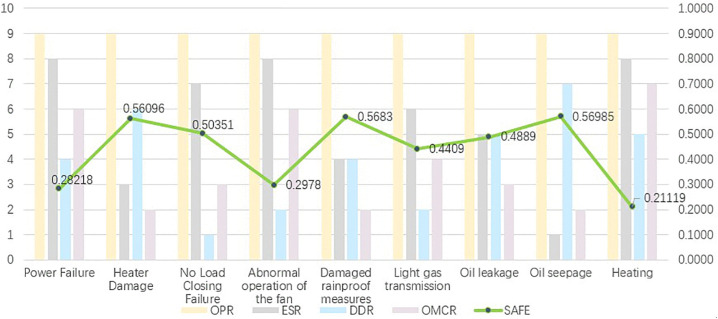
Safety factor results.

The expert scoring method is used to determine the priority of risks. Experts from Inner Mongolia Electric Power Company rated the severity and detection difficulty of each failure mode. Finally, on the basis of the risk priority calculated for each failure mode, the previous formula is used to calculate the safety factor. The calculated safety factors are shown in Fig 8.

Based on the safety factor in Fig 8 and combined with other parameters, obtain the maintenance strategy corresponding to each failure mode. The specific results are shown in the Table 15 below:

**Table 15:** Maintenance strategy results table.

Failure mode	Classification of maintenance strategy components	Ratio	M_annual_: the total annual maintenance frequency
Power failure	Emergency	10.2%	8.31
	Predictive	71.3%	
	Condition	16.8%	
	Time	1.6%	
Heater damage	Emergency	2.1%	6.63
	Predictive	33.2%	
	Condition	54.5%	
	Time	10.2%	
No-load closing failure	Emergency	4.5%	6.98
	Predictive	47.1%	
	Condition	39.8%	
	Time	8.6%	
Abnormal operation of the fan	Emergency	11.9%	8.21
	Predictive	67.6%	
	Condition	19.1%	
	Time	1.4%	
Damaged rainproof measures	Emergency	2.2%	6.59
	Predictive	25.1%	
	Condition	46.3%	
	Time	26.3%	
Light gas transmission	Emergency	6.8%	7.35
	Predictive	55.1%	
	Condition	33.8%	
	Time	4.3%	
Oil leakage	Emergency	3.5%	7.07
	Predictive	43.3%	
	Condition	45.8%	
	Time	7.5%	
Oil seepage	Emergency	1.6%	6.58
	Predictive	25.3%	
	Condition	62.2%	
	Time	10.9%	
Heating	Emergency	16.2%	8.73
	Predictive	75.2%	
	Condition	8%	
	Time	0.6%	

The results reflected in the table above can be summarized as follows:

1) The predictive maintenance weight corresponding to the four failure modes of Heating, Power failure, Abnormal fan operation, and Light gas transmission is greater than 50%. Because the consequences of these failure modes are severe, such as casing explosions and power outages. Fault symptoms must be detected 2–4 weeks in advance through proactive prediction, and preventive measures must be taken immediately. We cannot wait until we see the problem before dealing with it. Because the time window left for reaction is too short or the consequences are unacceptable.
2) The predictive maintenance and condition monitoring weights corresponding to the two failure modes of No-load closing failure and Oil leakage are close. These failure modes are located in the risk critical zone and require two strategies to be implemented in parallel. Ensure that neither progressive faults are missed nor sudden anomalies are captured.
3) The weight of the state monitoring strategy corresponding to the three failure modes of Oil seed page, Heater damage, and Damaged rainproof is greater than 50%. The core of strategy is to act after observation. Discover problems in a timely manner through low-cost and high-frequency inspections, and handle them before the fault expands. No need for expensive prediction systems.

**Fig 9:**
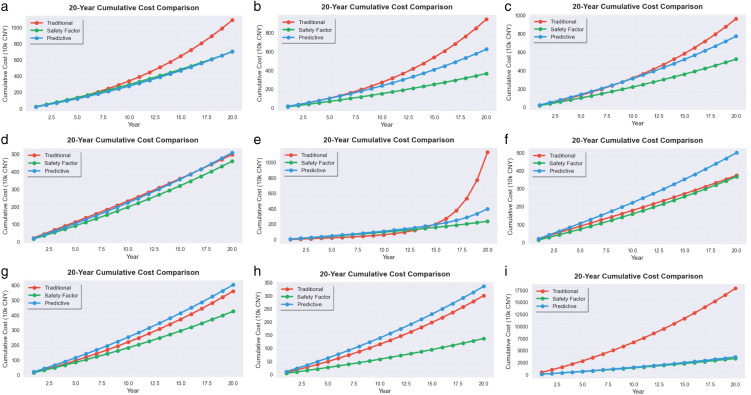
20 year cumulative cost comparison chart. (a) power failure; (b) heater damage; (c) no-load closing failure; (d)abnormal operation of the fan; (e) damaged rainproof measures; (f) light gas transmission; (g) oil leakage; (h) oil seepage; (i) heating.

**Fig 10:**
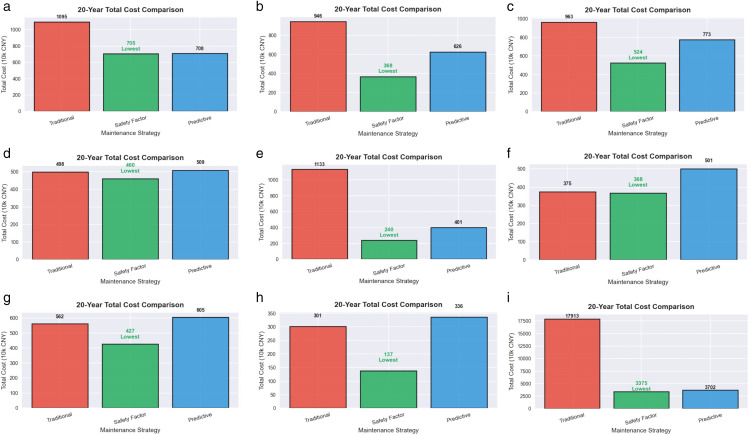
20 year total cost comparison chart. (a) power failure; (b) heater damage; (c) no-load closing failure; (d)abnormal operation of the fan; (e) damaged rainproof measures; (f) light gas transmission; (g) oil leakage; (h) oil seepage; (i) heating.

A series of cost data are obtained and compared with traditional scheduled maintenance strategies and pure predictive maintenance strategies. The specific results are shown in Fig 9 and Fig 10:

Fig 9 and Fig 10 show that the safety factor dynamic maintenance strategy has achieved significant economic advantages of the lowest total cost and a cumulative cost savings of 40–60% over 20 years. Compared to traditional scheduled maintenance strategies, the strategy proposed in this article can save an average of 43.36% of the total cost. Compared to predictive maintenance strategies, it can also save an average of 27.52% of total costs. This is mainly because the strategy utilizes a risk quantification mechanism based on RPN and learning curve effects. Fig 9 and Fig 10 fully verify the universality and economic effectiveness of the safety factor dynamic maintenance strategy.

It is worth noting that cost differentiation mainly occurs after 5 years of operation and significantly expands during the period of 12–20 years, fully verifying the long-term economic value and learning curve effect of the safety factor dynamic maintenance strategy.

**Fig 11:**
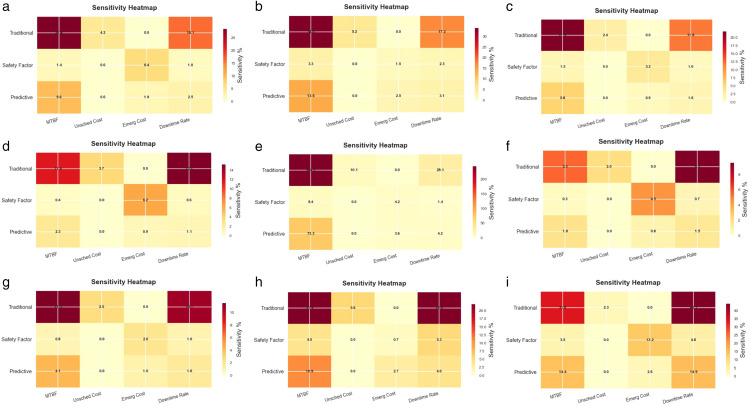
Sensitivity heatmap chart. (a) power failure; (b) heater damage; (c) no-load closing failure; (d)abnormal operation of the fan; (e) damaged rainproof measures; (f) light gas transmission; (g) oil leakage; (h) oil seepage; (i) cheating.

Finally, a ± 30% fluctuation analysis was conducted on four key parameters (MTBF, unscheduled maintenance cost, emergency maintenance cost, and downtime loss rate), as shown in the Fig 11. From Fig11, it can be concluded that the safety factor dynamic maintenance strategy can demonstrate significant decision robustness advantages in almost all failure modes. This fully validates its risk resistance ability in parameter uncertainty environments. Apart from the dimension of emergency maintenance cost. There is a phenomenon of strategy reversal in this dimension, and the sensitivity of the safety factor strategy is slightly higher than that of traditional maintenance. This anomaly is attributed to the presence of the emergency maintenance weight $w_{emergency}=\alpha_{1}\times {\left(1-SAFE\right)}^{2}$ in the dynamic mechanism, which results in a higher proportion of emergency costs in high-risk failure modes. Traditional maintenance, on the other hand, exhibits insensitive characteristics to this parameter due to the lack of differentiated response mechanisms. This discovery reveals the cost structure characteristics of dynamic maintenance strategies for safety factors. Although the proportion of emergency maintenance costs is low, it has become the main driving factor for cost fluctuations due to the dynamic weight allocation mechanism. This provides a quantitative basis for enterprises to develop differentiated cost control strategies. Priority should be given to reducing the unit price of emergency maintenance, rather than the cost of failure maintenance that traditional strategies focus on.

## 5. Conclusion

A transformer maintenance strategy based on an improved risk assessment method considering operation and maintenance costs is proposed to address the lack of consideration of failure modes in transformer reliability research and the incomplete applicability of traditional risk assessment methods to transformers. First, considering the impact of operation and maintenance costs on the formulation of transformer maintenance strategies, the traditional risk priority method is improved to include operation and maintenance costs as separate risk assessment factors. Starting from the failure mode, the risk assessment results are calculated by integrating operational data, maintenance records, and expert experience with transformers. On the basis of the risk assessment results, the safety factor and maintenance strategy are finally obtained. This research supplements traditional risk analysis methods, and the important impacts of cost factors on maintenance strategies are clarified; moreover, maintenance strategies are optimized on the basis of actual situations, thereby effectively guiding transformer maintenance and operation decisions. The results of this research indicate that by independently considering the impact of operation and maintenance costs on failure mode risk, economic-oriented failure mode recognition results and optimized maintenance strategies can be obtained. These findings can be used for the rational allocation of maintenance resources and to ensure the economy and safety of transformer systems. The proposed approach as high application value in practical operation and maintenance management work.

Potential future studies related to this work are as follows. First, beyond cost, additional risk assessment factors that may affect maintenance strategies should be considered. In addition, the research subject can be further expanded to other types of transformers, if relevant data can be obtained.
